# Treatment of Delayed Cerebral Ischemia in Good-Grade Subarachnoid Hemorrhage: Any Role for Invasive Neuromonitoring?

**DOI:** 10.1007/s12028-020-01169-x

**Published:** 2020-12-10

**Authors:** Michael Veldeman, Walid Albanna, Miriam Weiss, Catharina Conzen, Tobias Philip Schmidt, Hans Clusmann, Henna Schulze-Steinen, Omid Nikoubashman, Yasin Temel, Gerrit Alexander Schubert

**Affiliations:** 1 Department of Neurosurgery, RWTH Aachen University Hospital, Pauwelsstrasse 30, 52074 Aachen, Germany; 2 Department of Neurosurgery, Maastricht University Medical Centre, Maastricht, The Netherlands; 3 Department of Intensive Care Medicine, RWTH Aachen University, Aachen, Germany; 4 Department of Diagnostic and Interventional Neuroradiology, RWTH Aachen University, Aachen, Germany

**Keywords:** Subarachnoid hemorrhage, Invasive neuromonitoring, Brain tissue oxygen, Cerebral microdialysis, Delayed cerebral ischemia

## Abstract

**Background:**

Good-grade aneurysmal subarachnoid hemorrhage (Hunt and Hess 1–2) is generally associated with a favorable prognosis. Nonetheless, patients may still experience secondary deterioration due to delayed cerebral ischemia (DCI), contributing to poor outcome. In those patients, neurological assessment is challenging and invasive neuromonitoring (INM) may help guide DCI treatment.

**Methods:**

An observational analysis of 135 good-grade SAH patients referred to a single tertiary care center between 2010 and 2018 was performed. In total, 54 good-grade SAH patients with secondary deterioration evading further neurological assessment, were prospectively enrolled for this analysis. The cohort was separated into two groups: before and after introduction of INM in 2014 (pre-INM_SecD_: *n* = 28; post-INM_SecD_: *n* = 26). INM included either parenchymal oxygen saturation measurement (p_ti_O_2_), cerebral microdialysis or both. Episodes of DCI (p_ti_O_2_ < 10 mmHg or lactate/pyruvate > 40) were treated via induced hypertension or in refractory cases by endovascular means. The primary outcome was defined as the extended Glasgow outcome scale after 12 months. In addition, we recorded the amount of imaging studies performed and the occurrence of silent and overall DCI-related infarction.

**Results:**

Secondary deterioration, impeding neurological assessment, occurred in 54 (40.0%) of all good-grade SAH patients. In those patients, a comparable rate of favorable outcome at 12 months was observed before and after the introduction of INM (pre-INM_SecD_ 14 (50.0%) vs. post-INM_SecD_ 16, (61.6%); *p* = 0.253). A significant increase in good recovery (pre-INM_SecD_ 6 (50.0%) vs. post-INM_SecD_ 14, (61.6%); *p* = 0.014) was observed alongside a reduction in the incidence of silent infarctions (pre-INM_SecD_ 8 (28.6%) vs. post-INM_SecD_ 2 (7.7%); *p* = 0.048) and of overall DCI-related infarction (pre-INM_SecD_ 12 (42.8%) vs. post-INM_SecD_ 4 (23.1%); *p* = 0.027). The number of CT investigations performed during the DCI time frame decreased from 9.8 ± 5.2 scans in the pre-INM_SecD_ group to 6.1 ± 4.0 (*p* = 0.003) in the post-INM_SecD_ group.

**Conclusions:**

A considerable number of patients with good-grade SAH experiences secondary deterioration rendering them neurologically not assessable. In our cohort, the introduction of INM to guide DCI treatment in patients with secondary deterioration increased the rate of good recovery after 12 months. Additionally, a significant reduction of CT scans and infarction load was recorded, which may have an underestimated impact on quality of life and more subtle neuropsychological deficits common after SAH.

**Electronic supplementary material:**

The online version of this article (10.1007/s12028-020-01169-x) contains supplementary material, which is available to authorized users.

## Introduction

Aneurysmal subarachnoid hemorrhage (SAH) presents with an overall annual incidence of 9.1/100.000 patients [1, 2]. Surviving the initial hemorrhage, clinical outcome is further compromised by the occurrence of delayed cerebral ischemia (DCI) [3]. Cumulating evidence from clinical and laboratory research indicates that radiographic vasospasm is not the lone standing culprit of neurological deterioration, which is typically observed in SAH patients between day 4 and 14 after the initial bleed. Today, it is clear that macroscopic vasospasm is a contributing factor to the complex multifactorial pathological process of DCI [4, 5] including cortical spreading depolarization, microvascular spasms, formation of microthrombi and dysfunctional autoregulation [6–9].

A multitude of scales has been proposed to clinically estimate both the severity of aneurysmal SAH, but also the risk of complications such as DCI. The Hunt and Hess (HH) scale and WFNS scale are the most commonly used [10, 11]. Both scales are based on the clinical condition at time of presentation and correlate with the incidence of DCI, DCI-related cerebral infarction and long-term clinical outcome [12–14].

Patients presenting with mild to severe headache and an unaltered level of consciousness are typically graded as H&H grade 1 and 2 hemorrhages, with a lower risk of DCI and favorable prognosis. Nevertheless, once patients suffer secondary deterioration rendering them unconscious, DCI progression and treatment can no longer be monitored clinically [15]. A consensus clinical definition for DCI as neurological deterioration exists [16], but no consensus exists how to monitor the unconscious patient. Invasive neuromonitoring (INM) may help to identify additional waves of DCI events and offer a mean of continuous bed-side treatment surveillance. In 2014, INM—brain tissue oxygen monitoring (p_ti_O_2_) and cerebral microdialysis (CMD)—were introduced to our institutional treatment algorithm. We previously demonstrated that INM can be used to measure treatment efficacy of endovascular rescue strategies [17, 18] and can also accelerate the diagnosis of DCI events in poor-grade SAH patients, resulting in an earlier initiation of DCI treatment, ultimately improving overall outcome [19]. Moreover, INM led to a reduction of imaging, thus reducing radiation exposure and the need for potentially harmful transportation of critically ill patients [20].

In good-grade SAH patients, the initial DCI event is usually diagnosed clinically. If these patients become unconscious, further clinical monitoring beyond the initial deterioration is no longer feasible. INM as a means to quantify cerebral metabolism and/or oxygenation may aid in guiding treatment and monitor treatment effects. The purpose of this observational trial is to assess the impact of INM on the clinical course and outcome of good-grade SAH patients who experience secondary deterioration.

## Materials and Methods

### Patient Population and Study Design

This trial was designed as an observational cohort study and constitutes a subgroup analysis of partly previously published data [19]. The study was registered (NCT02142166) and approved by the ethics committee of the Medical Faculty of the RWTH Aachen University (EK 062/14). Consecutive patients with confirmed aneurysmal SAH referred to a single university hospital between 2010 and 2018 were screened for eligibility. Good-grade SAH patients (H&H 1–2) between 18 and 90 years of age were included. To correct for post-ictal reduced consciousness and to reduce interobserver variability, grading was allocated as the best clinical grade within 24 h after admission [21]. Cases with early mortality (< 4 days) or patients with mycotic or traumatic/pseudo aneurysms and aneurysms associated with arteriovenous malformations were excluded. Application of INM was considered in patients with secondary deterioration, impeding further neurological assessment. The exact cause of deterioration, e.g., DCI, untreated hydrocephalus, rebleeding, infection, electrolyte imbalance, seizure or infection, was identified. The cohort was then split into two subgroups, before and after introduction of INM in 2014 (pre-INM_SecD_, post-INM_SecD_). Blood pressure data (RR_syst_ and MAP) for both groups and intracranial pressure (ICP) as well as CPP data for the monitored group, were recorded hourly. Overall disease severity was scored during the first 24 h after admission using the APACHE II and SAPS II scores.

### Standard Treatment Algorithm

The diagnostic and therapeutic treatment algorithm at our institution has been published previously [17, 18]. In summary, aneurysms were secured by surgical clipping or endovascular coiling within 48 h after diagnosis of the hemorrhage. Each patient was observed for a minimum of 14 days on our neurointensive care unit. As good-grade SAH patients initially present awake and oriented, clinical assessment can detect DCI reliably, obviating the need for INM. The criteria set by Vergouwen et al. were used to define classical/clinical DCI: new focal neurologic deficit or a decrease in GCS ≥ 2 for at least 1 h, not ascribable to alternative diagnoses [16]. The diagnosis of secondary deterioration was made when patients deteriorated to a level where further clinical assessment is evaded. Beginning in 2014, invasive neuromonitoring probe placement (INM) was considered in those unconscious patients, observing the following relative contraindications known coagulation disorders (preexisting or iatrogenic) or predictable early mortality (brain stem damage on imaging or bilateral fixed pupils). Brain tissue oxygen probes (Neurovent PTO, Raumedic, Helmbrechts, Germany) and CMD catheters (71 High Cut-Off Brain Microdialysis Catheter, M dialysis, Stockholm, Sweden) were implanted ipsilateral of the side of the ruptured aneurysm or on the side with higher subarachnoid blood load in case of midline aneurysms. In order to cover two vascular territories (middle and anterior cerebral artery), probes were placed in the frontal watershed region, 4.5 cm lateral to the midline in front of the coronal suture. Brain partial oxygen pressure (mmHg) was recorded continuously, and interstitial glucose levels (mmol/l) and the lactate to pyruvate ratio (LPR) were registered every second to third hour.

**Fig. 1:**
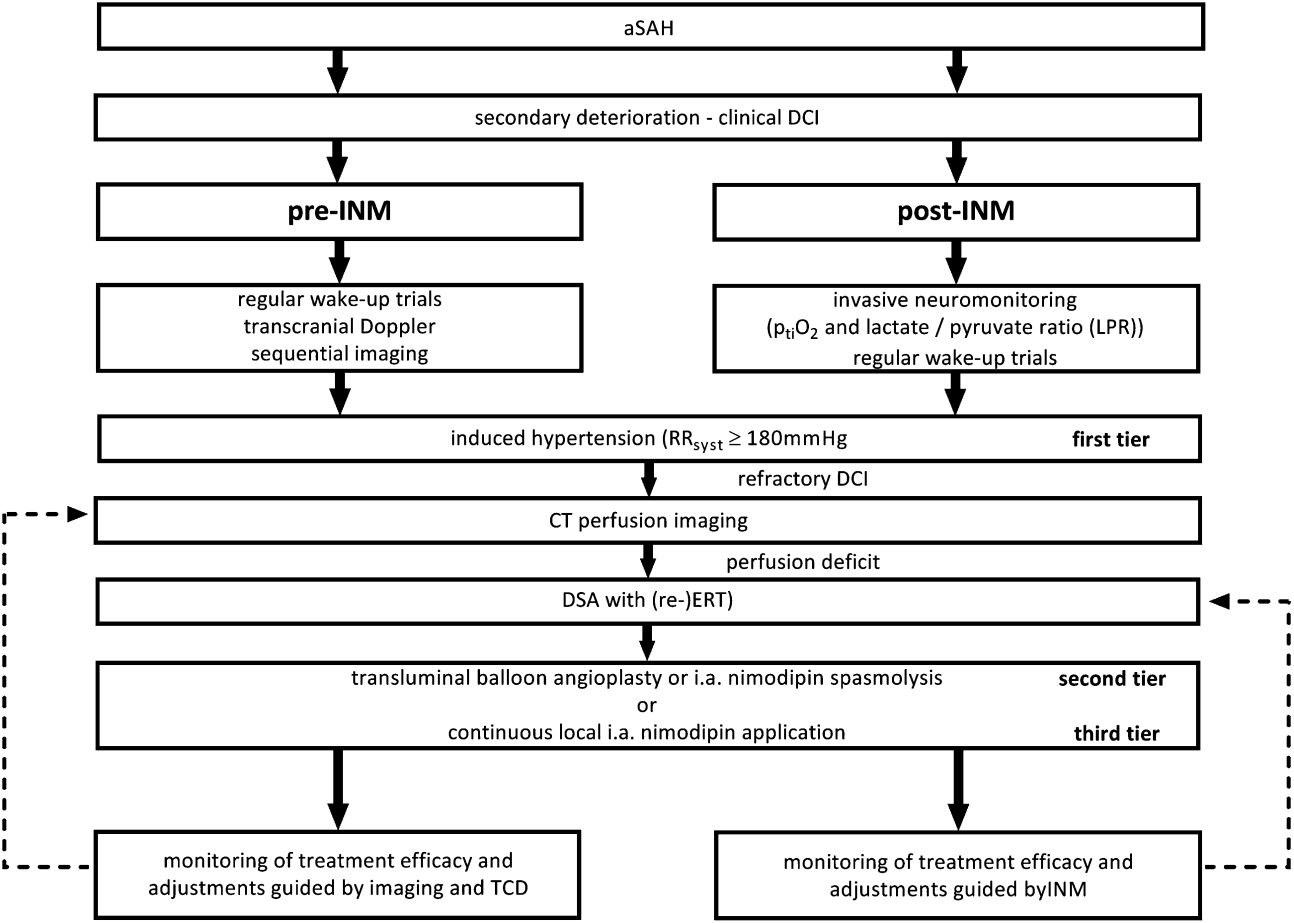
Differences in diagnostics, but identical treatment algorithm for the pre-INM and post-INM groups with secondary deterioration. In the pre-INM group, induced hypertension was guided by TCD and sequential imaging. In the post-INM group, DCI treatment was guided by INM and INM-triggered imaging. This included the timing and further monitoring of second and third tier endovascular treatment. DCI, delayed cerebral ischemia; DSA, cerebral digital subtraction angiography; ERT, endovascular rescue therapy; i.a., intra-arterial; INM, invasive neuromonitoring; RR, blood pressure; TCD, transcranial Doppler

After the initial clinical DCI diagnosis, first tier treatment consisted of euvolemic arterial hypertension (≥ 180 mmHg) via intravenous norepinephrine infusion. As soon as INM was in place, DCI treatment was guided by eversion of oxygenation crises (p_ti_O_2_ < 10 mmHg) [22] or metabolic derangements (LPR ≥ 40) [23] with iHTN. In cases, refractory to hypertensive treatment CT-perfusion imaging was performed for re-evaluation. When perfusion mismatch persisted, despite induced hypertension, second tier endovascular rescue therapy including intra-arterial spasmolysis and balloon angioplasty was considered. The effect and duration of resolution were quantified and monitored via INM as described previously [17]. The treatment algorithm is depicted in Fig. 1.

### Outcome Definition

The primary outcome was defined as the extended Glasgow outcome scale (GOSE) 12 months after the initial hemorrhage and was recorded in all patients [24]. The majority of outcome data was collected prospectively during regular follow-ups, but missing information was either appended by analysis of medical records or a structured telephone interview by a blinded assessor [25]. In the pre-INM group, in many cases, the GOSE at 12 months had to be reconstructed retrospectively by investigation of medical records or by contacting the patient, his next of kin, or caregiver in a structured telephone interview. Patients were categorized into one of eight outcome grades. As is conventional, the GOSE scale was dichotomized into unfavorable (GOSE_1–4_) and favorable (GOSE_5–-8_) outcome. The secondary outcomes were predefined as: the number of CT investigations performed during the DCI time frame, the prevalence of silent infarctions (the diagnosis of a demarcated infarction as a first sign of ongoing DCI), the incidence of overall DCI-related infarction and DCI-related mortality. DCI-related mortality was defined as the withdrawal of technological life support due to severe DCI-induced damage. An additional aim was to identify predictors of secondary deterioration related to DCI, in good-grade SAH. Finally, also the absolute changes of INM measurements before and after DCI treatment were assessed.

### Statistical Analysis

Predictors of secondary deterioration in patients diagnosed with DCI, were identified with a binomial logistic regression model. Continuous covariates were tested for linearity via a Box–Tidwell assessment. Predictor covariates were included when a *p* < 0.1 was identified in univariate testing.

The primary endpoint was analyzed in an ordinal logistic regression model with the mFisher, HH grade and age as predictor covariates. The model was accepted in case of a nonsignificant test result of the proportional odds assumption. Additionally, dichotomous GOSE outcome data were compared between groups using chi-square testing. Data are presented as the mean and standard deviation for continues variables unless stated otherwise. Categorical variables are presented as frequencies and proportions. After normality testing via the Shapiro–Wilk test, the appropriate statistical test was selected. Nominal data were tested with the chi-square test, for normally distributed continuous data the unpaired *t*-test and for non-normal distributed data the Mann–Whitney *U*-test were used. Continuous longitudinal data were compared using a mixed-effects model using a restricted maximum likelihood approach with Greenhouse–Geisser correction, as an alternative to a two-way ANOVA, due to occasional missing values. Missing data were not imputed. All statistical analyses were performed using IBM SPSS Statistics 25 (SPSS Inc., Chicago, IL, USA), and graphics were plotted using GraphPad Prism 8.1.1 (GraphPad Software, Inc., La Jolla, CA, USA). Statistical significance was defined as a two-sided *p* > 0.05. All analyses were carried out on an intention-to-treat and per-protocol basis.

## Results

### Patients

**Fig. 2:**
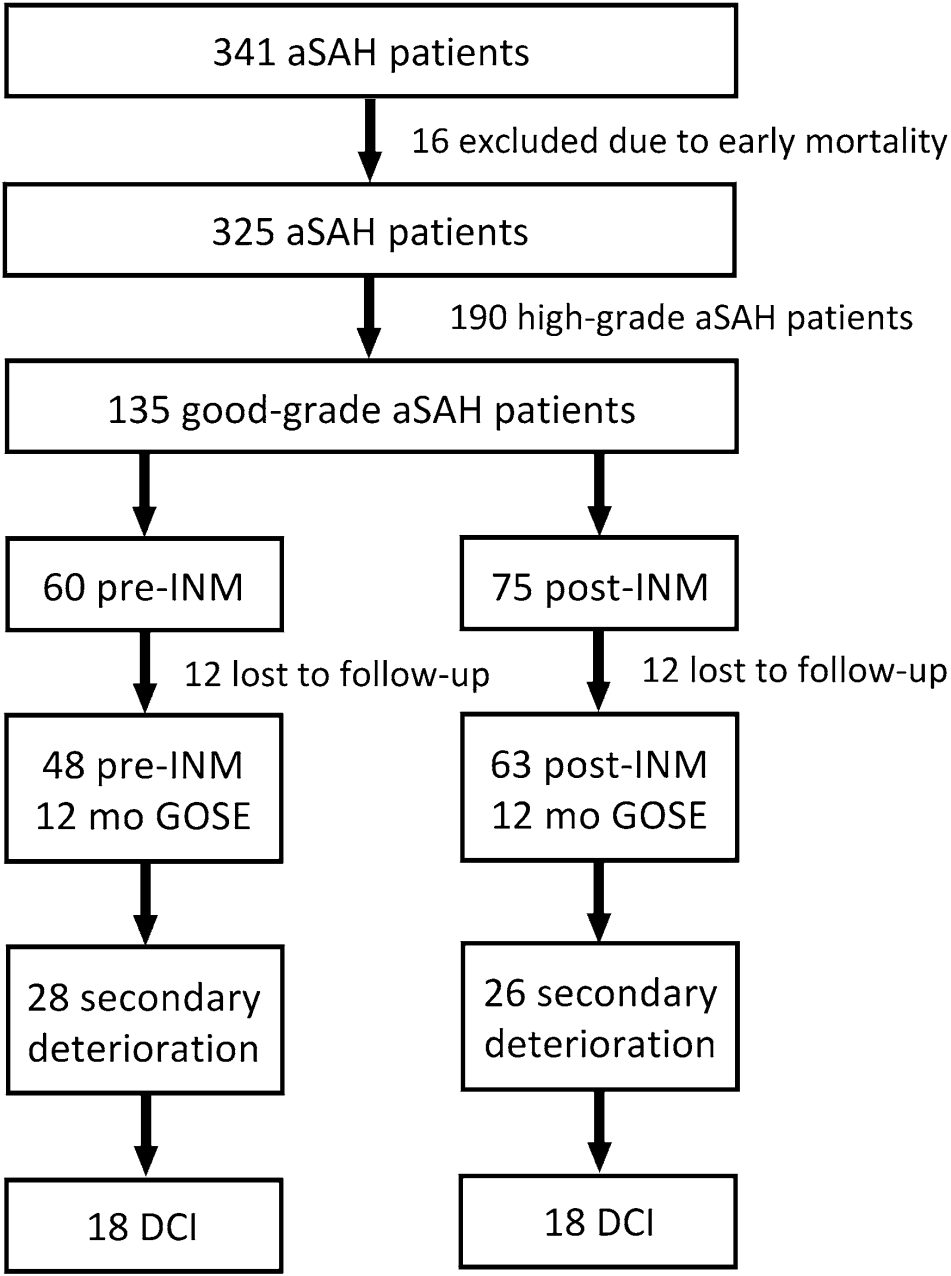
Flowchart of patient enrollment

The recruitment process is depicted in Fig. 2. A total of 341 SAH patients were treated at our institution between 2010 and 2018. Sixteen patients were excluded due to early mortality. Of the remaining patients, 135 were classified as Hunt and Hess grade 1 or 2. Of those, 60 were included prior to implementation of invasive neuromonitoring (pre-INM) and 75 after (post-INM). In each group, 12 patients were lost to follow-up resulting in missing outcome data. None of these patients suffered secondary deterioration during their ICU stay. This left 111 patients to be included in our study. Baseline characteristics of the whole cohort are presented in Suppl. Table 1. No significant differences in outcome relevant baseline characteristics were noted (e.g., length of ICU stay, infection rate, hydrocephalus, etc.). Of the remaining cases, secondary deterioration occurred in 28 patients (58.3%) in the pre-INM group (pre-INM_SecD_) and 26 (41.3%) cases in the post-INM group (post-INM_SecD_). These 54 patients were included in our subgroup analysis. Of those 44 cases required intubation to ensure adequate airway protection. Patients in the pre-INM_SecD_ group were monitored by TCD, periodic imaging and repeated wake-up trials. All patients with deterioration in the post-INM_SecD_ group received INM consisting of a p_ti_O_2_ probe, a microdialysis probe or both (*n* = 17; 43 probes in total). Of all patients with secondary deterioration, 18 (64.3%) in the pre-INM_SecD_ and 18 (69.2%) in the post-INM were diagnosed with clinical DCI due to a GCS drop with or without a neurologic deficit and this after exclusion of other reversible causes, i.e., hydrocephalus, seizure, electrolyte imbalance, etc. Relevant baseline characteristics of both groups are summarized in Table 1 and were comparable, with the exception of a significant shift toward endovascular aneurysm treatment in the post-INM_SecD_ group (pre-INM_SecD_ 10 (35.7%) vs. post-INM_SecD_ 17 (65.4%); *p* = 0.028).

**Table 1:** Baseline characteristics of both groups

Characteristic	pre-INM (*n* = 28)	INM (*n* = 26)	*p* value
Age-years.—mean ± SD (range)	60.1 ± 13.3 (28–82)	58.7 ± 11.6 (32–80)	0.688
Gender: Female (%)/Male (%)	20 (71.4)/8 (28.6)	18 (69.2)/8 (30.8)	0.548
*Risk factors—no. (%)*			
Hypertension	11 (39.3)	12 (46.2)	0.407
Smoking	7 (25.0)	7 (26.9)	0.558
BMI	23.7 ± 4.1	26.0 ± 5.0	0.078
DM2	1 (3.6)	1 (3.8)	0.736
Alcohol abuse	2 (7.1)	1 (3.8)	0.528
Aneurysm diameter max. (mm)	6.8 ± 3.5	7.5 ± 2.7	0.187
Hunt and Hess grade - no. (%)			0.462
Grade 1	18 (64.3)	18 (69.2)	
Grade 2	10 (35.7)	8 (28.6)	
Aneurysm location—no. (%)			0.316
MCA	7 (25.0)	3 (11.5)	
Acom	8 (28.6)	15 (57.7)	
ICA	3 (10.7)	1 (3.8)	
Pcom and AchA	3 (10.7)	2 (7.8)	
BA	4 (14.3)	0 (0.0)	
Others	3 (10.7)	5 (19.2)	
Anterior circulation	23 (82.1)	24 (92.3)	
Posterior circulation	5 (17.9)	2 (7.7)	
Clipping/Coiling	18 (64.3)/10 (35.7)	9 (34.6)/17 (65.4)	**0.028**
Hydrocephalus	17 (60.7)	20 (77.0)	0.200
Modified Fisher grade			0.373
Grade 1	8 (28.6)	7 (26.9)	
Grade 2	3 (10.7)	5 (19.2)	
Grade 3	14 (50.0)	4 (15.4)	
Grade 4	3 (10.7)	10 (38.5)	

Statistically significant value is given in bold at *p* < 0.05

There was a significantly larger proportion of aneurysms treated by endovascular coiling in the post-INM group. The pre-INM group contained mostly mFisher grade 3 patients, whereas mFisher grad 4 made up the larger proportion of the post-INM group. In an ordinal regression model, there was no significant difference in the groups mFishers´ distribution. No other relevant differences in baseline characteristics were noted

*AchA* anterior choroidal artery, *Acom* anterior communicating artery, *APACHE II* Acute Physiology and Chronic Health Evaluation II score, *BA* basilar artery, *BMI* body mass index, *CMD* cerebral microdialysis, *DM2* type 2 diabetes, *ICA* internal cerebral artery, *INM* invasive neuromonitoring, *MCA* middle cerebral artery, *Pcom* posterior communication artery, *p*_*ti*_*O*_*2*_ brain tissue oxygen monitoring, *SAPS II* simplified acute physiology score

The algorithm for DCI treatment remained unchanged from 2010 to 2018. DCI occurred on average 6.7 ± 4.0 days after ictus in the pre-INM_SecD_ group and after 7.7 ± 2.9 days in the post-INM_SecD_ group (*p* = 0.467), triggering the induction of euvolemic hypertension (iHTN). Endovascular rescue treatment was applied due to refractory DCI in 10 patients in both groups corresponding to 35.7% of DCI patients in the pre- *vs.* 38.5% in the post-INM_SecD_ groups (*p* = 0.704). (Suppl. Table 2.) In the post-INM_SecD_ group, INM was used to guide iHTN treatment and helped to identify refractory cases eligible for ERT. In the pre-INM_SecD_, this was solely based on TCD results and repetitive imaging.

### Complications

With 43 probes implanted, a total of 5 hemorrhages were detected on postoperative imaging. Two cases featured a small epidural hematoma and three cases showed blood along the tract of the probe. This corresponds to a 11.6% risk per probe placement. None required additional surgical intervention, and no permanent neurological sequelae were observed. Two of the hemorrhages along the tract of the probe occurred under dual antiplatelet treatment in the context of complicated coiling. One hemorrhages occurred during probe removal while performing a decompressive hemicraniectomy. Three patients (11.5%) developed positive cultures of CSF collected from a ventriculostomy. The contribution of invasive probes to CSF contamination is difficult to estimate. It is worth noting that no probes required removal due to overt signs of infection.

### Baseline Group Comparison

**Fig. 3:**
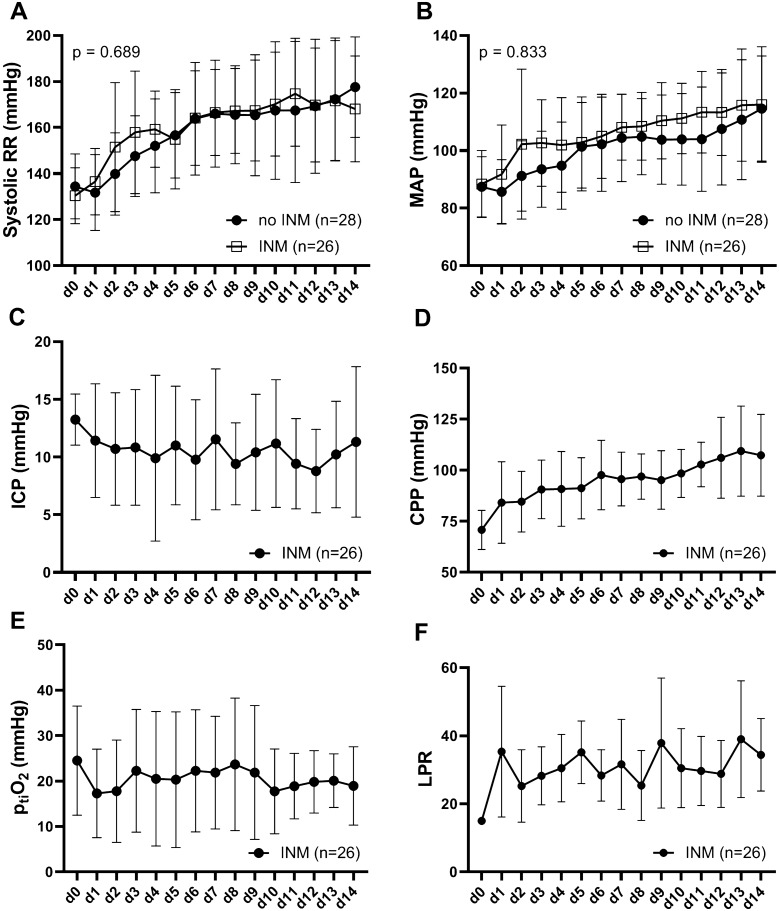
Graphic representation of systolic blood pressure (**a**) and MAP (**b**) in both groups. ICP (**c**) and CPP (**d**) measurements were only available in the monitored groups. Brain tissue oxygen levels (**e**) and the lactate to pyruvate ratio were also plotted for the first 14 post-hemorrhage days. CPP, cerebral perfusion pressure; ICP, intracranial pressure; LPR, lactate to pyruvate ratio; MAP, mean arterial pressure; p_ti_O_2_, brain tissue oxygen pressure; RR, blood pressure

Both groups proofed comparable for demographics (age and gender), SAH specific (H&H and mFischer) and general disease severity (APACHE II and SAPS II) characteristics. (Table 1) The course of systolic blood pressure (*F* (14, 589) = 0.782; *p* = 0.689) and MAP (*F* (14, 614) = 0.640; *p* = 0.833) did not differ when looking at the first 14 post-hemorrhage days and was plotted in Fig. 3a, b. ICP and calculated CPP were only available in the post-INM_SecD_ and are depicted in Fig. 3c, d.

### Predictors of Secondary Deterioration and Outcome

A binomial logistic regression model was performed for all patients, to ascertain the effects of age, gender, aneurysm treatment modality, Hunt and Hess and modified Fisher grading and hydrocephalus. Before model fitting, multicollineary was identified between mFisher grading and hydrocephalus and therefore, of the two, only mFisher was introduced into the analysis. The model was significant, (*χ*^2^(5) = 23.471; *p* < 0.001) and explained 21.6% (Nagelkerke *R*^*2*^) of the variance in occurrence of DCI-related secondary deterioration. Of the 5 predictor variables, only the modified Fisher (OR = 1.882; 95% CI 1.276–2.776; *p* = 0.001) and age (OR = 1.033; 95% CI 1.001–1.065; *p* = 0.043) were associated with an increased likelihood of developing secondary deterioration.

Due to significant differences in aneurysm occlusion modality between groups, clipping versus coiling was introduced into a binomial regression model, including all of the above predictors, assessing the effect on dichotomized GOSE. Aneurysm treatment modality had no effect on the occurrence of favorable or unfavorable outcome after 12 months (OR = 2.142; 95% CI 0.588–7.804; *p* = 0.248). Only the modified Fisher grading came close to having a significant effect on outcome (OR = 0.532; 95% CI 0.279–1.013; *p* = 0.055).

### Primary Outcome Parameter

**Fig. 4:**
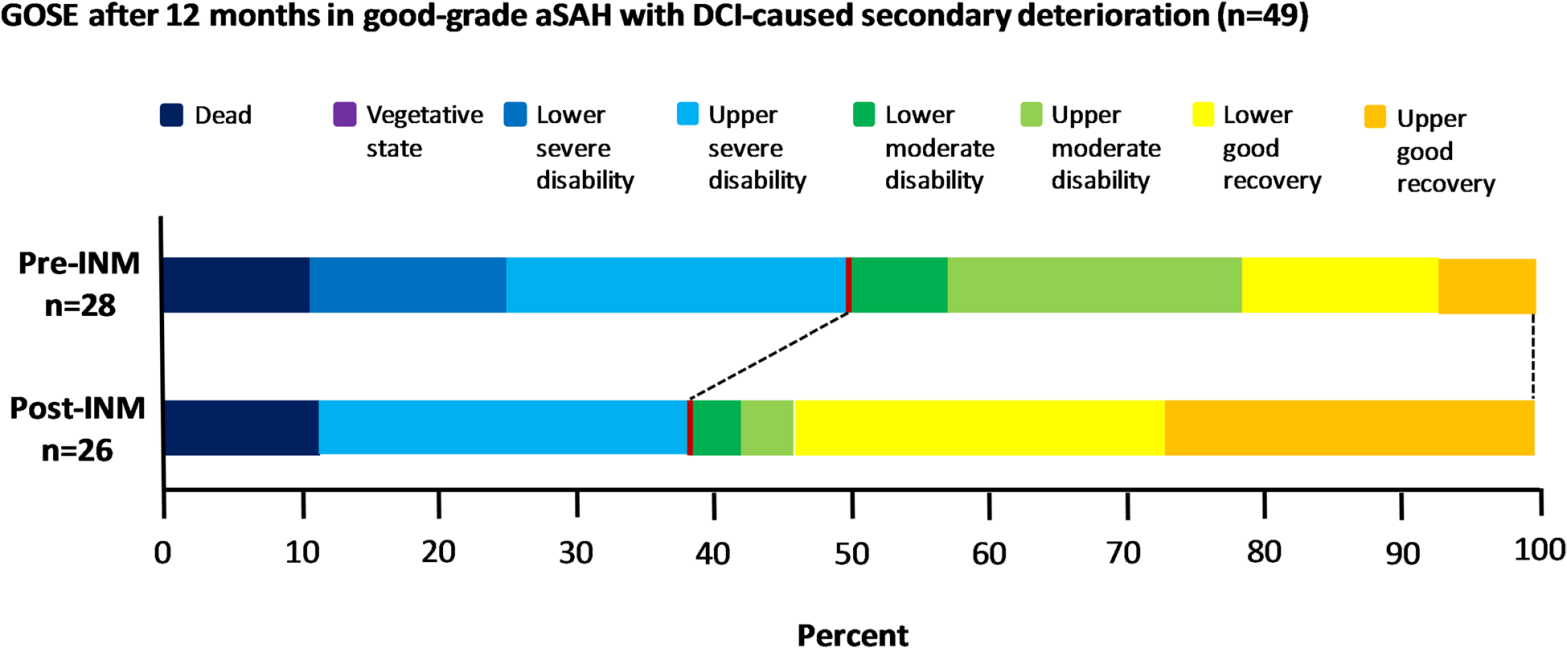
Stacked bar chart of extended Glasgow outcome scale (GOSE) results after 12 months in the pre-INM and post-INM groups with secondary deterioration

A high percentage of all good-grade SAH patients achieved favorable outcome after 1 year (*n* = 98, 88.3%). For patients with secondary deterioration, clinical outcome after 12 months was available in 54 cases. In this subgroup, favorable outcome was achieved only in 30 (55.6%) patients. The rate of favorable outcome (GOSE_5–8_) before and after introduction of INM did not differ significantly (14 (50.0%) vs. 16 (61.6%); *χ*^2^(1) = 1.305; *p* = 0.253). However, good recovery was more frequent after the introduction of INM, with of patients (20.4%) achieving lower and upper good recovery in the pre- and 14 (53.8%) patients in the post-INM_SecD_ group (GOSE_7–8_); *χ*^2^(1) = 6.075 (*p* = 0.014). In an ordinal logistic regression model, the odds ratio of being in a higher outcome grade based on INM application of 0.516 (95% CI, 0.172–1.548), did not show a significant effect *χ*^2^(1) = 1.394; *p* = 0.238 (Fig. 4).

### Secondary Outcome Parameters

**Fig. 5:**
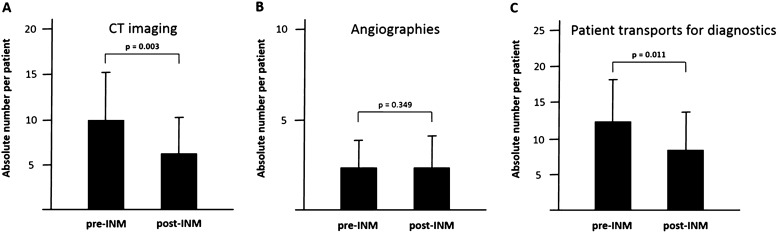
Bar chart of secondary outcome in the pre- and post-INM groups. Introduction of INM into our diagnostic algorithm resulted in a significant reduction of CT imaging (**a**), the number of angiographies per patients remained constant (**b**), but the overall number of transports per patients for diagnostic purposes was significantly reduced (**c**)

**Fig. 6:**
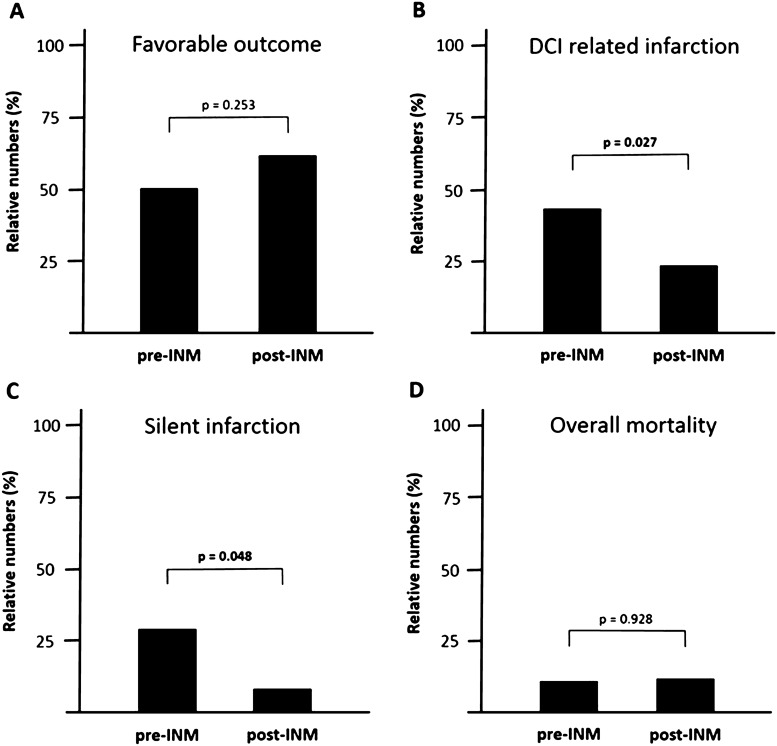
Bar charts of clinical and secondary outcome in the pre- and post-INM groups. Dichotomized clinical outcome data did not differ between the pre- and post-INM groups (**a**). Introduction of INM resulted in a significant reduction of DCI-related (**b**) and silent infarctions (**c**). The overall mortality remained unchanged between both groups (**d**). DCI, delayed cerebral ischemia

A significant reduction in the number of CT investigations performed during the DCI time frame was observed after INM implementation (9.8 ± 5.2 scans in the pre-INM_SecD_ group vs. 6.1 ± 4.0; *p* = 0.003), resulting in an overall decrease in patient transports (12.2 ± 5.8 vs. 8.2 ± 5.3; *p* = 0.011) (Fig. 5). There was a reduction in the incidence of silent infarctions (8 (28.6%) vs. 2 (7.7%); *p* = 0.048) and of overall DCI-related infarction (12 (42.8%) vs. 4 (23.1%); *p* = 0.027) (Fig. 6). There was no difference in overall (10.7% vs. 3.8%; 0.928) nor DCI-related (3 (10.7%) vs. 1 (3.8%); *p* = 0.336) mortality.

### Changes in INM Parameter After DCI Treatment

**Fig. 7:**
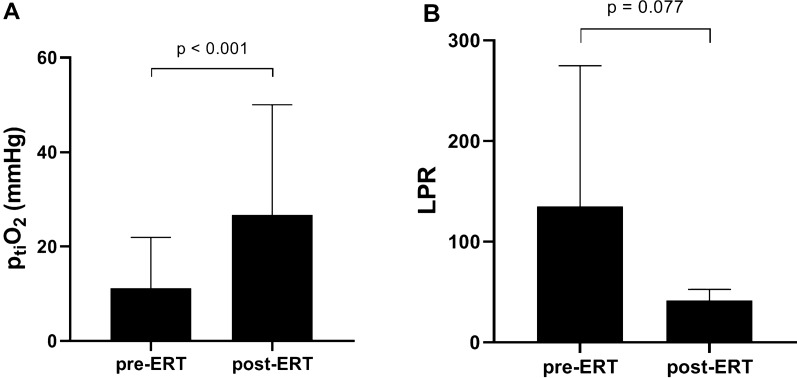
Changes in INM parameter in all patients treated with endovascular rescue treatment (*n* = 10). When comparing INM measurements collected 24 h prior to ERT with levels 24 h post-ERT, endovascular treatment induced a significant increase in p_ti_O_2_ from 5.8 ± 19.6 to 23.7 ± 32.2 (*p* < 0.001). The LPR showed a non-significant decrease from 135.0 ± 139.6 to 45.1 ± 11.3 (*p* = 0.777). ERT, endovascular rescue treatment; p_ti_O_2_, brain tissue oxygen pressure

The majority of patients with secondary deterioration received probes shortly before initiation of iHT. Therefore, the effect of iHT on individual INM registered parameter could not be evaluated in a meaningful manner. Hypertensive treatment was guided by those measurements, and refractory DCI was defined as a lack of p_ti_O_2_ and LPR response to iHT with confirmed persisting hypoperfusion on CTP imaging. ERT was applied in 10 of the post-INM_SecD_ cases. We compared INM measurements collected 24 h prior to ERT with levels 24 h post-ERT to evaluate the treatment effect of endovascular rescue treatment. Endovascular treatment induced a significant increase in p_ti_O_2_ from 5.8 ± 19.6 to 23.7 ± 32.2 (*p* < 0.001). The LPR showed a decrease from 135.0 ± 139.6 to 45.1 ± 11.3 (*p* = 0.777), albeit nonsignificantly in this small cohort of monitored cases. (Figure 7).

## Discussion

Earlier, goal-directed treatment guided by INM can facilitate an individualized treatment plan in poor-grade SAH patients, avoiding unnecessary diagnostic and therapeutic maneuvers. Vigilance for treatment response may also be higher with INM, and rescue treatment may be de-escalated earlier. The use of INM in good-grade SAH patients, however, is less clear.

Good-grade SAH patients can often expect a favorable outcome, but a considerable number of patients may still experience secondary deterioration due to DCI, which in turn is associated with worse outcome. In this observational cohort comparison, more than a third of good-grade SAH patients experienced secondary deterioration. Of those patients, only half still achieved favorable outcome, odds that are comparable to patients with poor-grade SAH [26]. This implies that DCI is of prognostic importance and not an infrequent observation in a presumably benign subgroup of SAH patients.

The focus of our diagnostic and treatment algorithm consists of brain tissue oxygenation and LPR values. Additional markers were simultaneously measured in the dialysate including glucose and glutamate. We acknowledge that for a full metabolic assessment, interstitial glucose levels are indispensable. However, as hyperglycemia can also be indicative for DCI, it remains unclear how to respond therapeutically to aberrant measurements, and therefore it was not taken into consideration in our diagnostic algorithm.

In our cohort, INM-guided DCI treatment of good-grade SAH patients with secondary deterioration resulted in a significantly higher rate of good recovery. When comparing the distribution of GOSE between both groups in an ordinal regression model, INM did not contribute to a difference in outcome distributions possible explained by a lack of statistical power based on the relative small sample size. Even in this small group, however, the implementation of INM resulted in a significant reduction of CT scans and transports. In-house transports are a well-known risk factor for additional complications [20], and in addition to a reduction in radiation exposure, fewer scans may very well improve the safety profile in those patients.

With regards to safety, we report a relatively high hemorrhagic complication rate of 11.6%. This has to be seen in light of the fact that every patient received post-implantation scanning and minimal hyperdensities on CT scan along the probe´s trajectory were counted as a hemorrhagic complication. Two out of five hemorrhages occurred under dual antiplatelet treatment emphasizing the need for probe implantation prior to stent-assisted coiling.

This is in line with our results that, the number of infarctions—silent and DCI-related—also decreased after implementation of INM while the number of CT scans and transportation could be reduced, implying that INM could be a beneficial and comparatively safe extension to our monitoring armamentarium in selected cases. Patients frequently report worse quality of life after SAH, as well as mood changes, depression and irritability, but also diminished ability to concentrate and perform more demanding intellectual tasks. Thorough neuropsychological testing is seldom performed, but usually shows significant impairment [27, 28], even years after SAH; those changes are usually not captured by GOSE assessment alone. We believe that the decrease in overall ischemic burden, as observed in our cohort with INM, may be reflected in the higher rate of good recovery and may very well have a positive impact on those more subtle changes and limitations patients often experience after SAH. It is an inherent limitation to our study and treatment protocol that routine neuropsychological assessment could not be implemented thus far.

We acknowledge also the limitations regarding the partially retrospective reconstructed outcome data, which is susceptible to recall bias. Moreover, we must address the fact, although the actual treatment algorithm apart from INM was not changed between both cohorts, the introduction of INM might be coalescent with a higher general surveillance of patients, independent of INM measurements and resulting treatment decisions.

In this cohort, probes were placed relatively late, in most cases after DCI-related deterioration had already set in. We believe that there exists a need for a better a priori assessment tool, to identify those good-grade SAH patients at higher risk of DCI development. Ideally, potential candidates are then identified earlier for monitoring implantation, before secondary deterioration occurs. A better risk stratification is necessary as scales based solely on the initial clinical grade have limited predictive value. Alternative grading scales have been proposed with mixed results. As it combines both the mFisher and WFNS scale, the VASOGRADE scale stratifies patients in three risk categories for DCI development [29]. However, with an area under the curve of 0.63 in the ROC analysis it remains a statistically poor predictor of DCI. The NIHSS scale was not better in predicting DCI and favorable outcome compared to the HH or mFisher scales [30].

In our cohort, every increase in mFisher grading was associated with a 1.87 times higher odds to exhibit later DCI-related deterioration. Additionally, age was an independent risk factor for DCI development and secondary deterioration. These results suggest the consideration of early implementation of invasive monitoring in older good-grade SAH patients with higher mFisher grading.

Neurosurgery in general is highly reliant on medical imaging. The comatose state of neurosurgical patients—being it trauma or SAH induced—poses a great challenge to the intensive care management of these cases. Multimodal monitoring probes constitute an alternative to sequential imaging to better guide treatment for complications such as DCI. On the other hand, one has to accept and understand the limitations of invasive monitoring. As it remains a local measurement covering only two unilateral vascular territories, contralateral or more posterior regions are not being surveilled. The reading of propitious values may induce a false feeling of assurance leading to missed treatment opportunities. The chance of covering the involved vascular territory in DCI development was discussed extensively by Ulrich et al. and this study confirms that due to the laterality of the single probe, midline aneurysms such as ACom an BA aneurysms, where DCI can occur on either side, make up a problematic subgroup [31]. Subsequently, technical problems and erroneous measurement remain an existing additional challenge when interpreting monitoring measurements.

## Conclusion

A considerable number of patients with good-grade SAH may experience secondary deterioration impeding further neurological assessment. The introduction of INM to guide DCI treatment in these patients, resulted in a reduction of CT scans and a lower rate of infarction. In this small cohort, an increase in patients with good recovery, was observes.

## Electronic Supplementary Material

Below is the link to the electronic supplementary material.

Supplementary Table 1. Relevant additional baseline characteristics of all good-grade SAH patients. (DOCX 13 kb)

Supplementary Table 2. Results comparing outcome and DCI treatment between the pre-INM and post-INM groups. The GOSE outcome categories were defined as follows: 1 = death, 2 = vegetative state (unable to obey commands), 3 = lower severe disability (dependent on others for care), 4 = upper severe disability (independent at home), 5 = lower moderate disability (independent at home and outside the home but with some physical or mental disability), 6 = upper moderate disability (independent at home and outside the home but with some physical or mental disability, with less disruption than lower moderate disability), 7 = lower good recovery (able to resume normal activities with some injury-related problems), and 8 = upper good recovery (no problems). CLINA, continuous local intra-arterial nimodipine application; DCI, delayed cerebral ischemia; ERT, endovascular rescue therapy; GOSE, Glasgow Outcome Scale; iHTN, induced hypertension; INM, invasive neuromonitoring. (DOCX 14 kb)

## Data Availability

The raw data of this analysis can be made available by the authors to any qualified researcher.

## Abbreviations

ACom: Anterior communicating artery
APACHE II: Acute Physiology and Chronic Health Evaluation II
BA: Basilar artery
CBF: Cerebral blood flow
CMD: Cerebral microdialysis
CLINA: Continuous local intra-arterial nimodipine application
CPP: Cerebral perfusion pressure
CSD: Cortical spreading depolarization
CSF: Cerebrospinal fluid
CT: Computed tomography
CTP: Perfusion CT imaging
DCH: Decompressive hemicraniectomy
DCI: Delayed cerebral ischemia
ERT: Endovascular rescue treatment
EVD: External ventricular drainage
GCS: Glasgow coma scale
GOSE: Extended Glasgow outcome scale
HH: Hunt and Hess grade
ICA: Internal carotid artery
ICP: Intracranial pressure
iHTN: Euvolemic induced hypertension
INM: Invasive neuromonitoring
MAP: Mean arterial pressure
MCA: Middle cerebral artery
mFisher: Modified fisher scale
p_ti_O_2_: Brain tissue oxygen pressure
RR: Blood pressure
SAH: Subarachnoid hemorrhage
SAPS II: Simplified Acute Physiology Score II
TCD: Transcranial Doppler sonography

## Trial Registration

This observational trial was retrospectively registered at the ISRCTN registry (Reference No. 16523175) on 07/25/2019. The prospective data collection was registered on 05/20/2014 at ClinicalTrials.gov (NCT02142166) as part of a larger scale prospective data collection.
