# Supplementary material for: Treatment of Delayed Cerebral Ischemia in Good-Grade Subarachnoid Hemorrhage: Any Role for Invasive Neuromonitoring?
Source: Neurocrit Care. 2020 Dec 10;35(1):172–83. doi: 10.1007/s12028-020-01169-x (PMC8285339; doi:10.1007/s12028-020-01169-x)
Supplement: Supplementary file 1 — Supplementary Table 1. Relevant additional baseline characteristics of all good-grade SAH patients. (DOCX 13 kb) [file 12028_2020_1169_MOESM1_ESM.docx]

| **Variable** | **pre-INM (n=96)** | **post-INM (n=94)** | **p-value** |
| --- | --- | --- | --- |
| **Baseline characteristics** |  |  |  |
| Multiple aneurysms - no. (%) | 22 (22.9) | 33 (35.1) | 0.164 |
| Initial seizure- no. (%) | 31 (32.3) | 19 (22.2) | 0.059 |
| Initial loss of consciousness- no. (%) | 64 (66.7) | 57 (60.6) | 0.388 |
| Intraventricular hemorrhage- no. (%) | 37 (38.5) | 34 (36.2) | 0.735 |
| Intracerebral hemorrhage- no. (%) | 43 (44.8) | 53 (56.4) | 0.190 |
| **ICU parameters** |  |  |  |
| ICU length of stay - days - mean ± SD | 25.3 ± 16.8 | 28.4 ± 17.5 | 0.166 |
| EVD | 70 (72.9) | 69 (73.4) | 0.965 |
| CSF infection | 11 (11.5) | 19 (20.2) | 0.098 |
| DCH | 27 (28.1) | 25 (26.6) | 0.943 |
| Tracheostomy - no. (%) | 34 (35.4) | 45 (47.9) | 0.071 |
| ICP crises - no. (%) | 30 (31.3) | 32 (34.0) | 0.573 |
| EVD duration - days - mean ± SD | 17.7 ± 11.1 | 20.1 ± 11.6 | 0.155 |
| CT imaging (no.) | 8.4 ± 5.3 | 6.8 ± 5.0 | **0.038** |
