# Supplementary material for: Treatment of Delayed Cerebral Ischemia in Good-Grade Subarachnoid Hemorrhage: Any Role for Invasive Neuromonitoring?
Source: Neurocrit Care. 2020 Dec 10;35(1):172–83. doi: 10.1007/s12028-020-01169-x (PMC8285339; doi:10.1007/s12028-020-01169-x)
Supplement: Supplementary file 2 — Supplementary Table 2. Results comparing outcome and DCI treatment between the pre-INM and post-INM groups. The GOSE outcome categories were defined as follows: 1 = death, 2 = vegetative state (unable to obey commands), 3 = lower severe disability (dependent on others for care), 4 = upper severe disability (independent at home), 5 = lower moderate disability (independent at home and outside the home but with some physical or mental disability), 6 = upper moderate disability (independent at home and outside the home but with some physical or mental disability, with less disruption than lower moderate disability), 7 = lower good recovery (able to resume normal activities with some injury-related problems), and 8 = upper good recovery (no problems). CLINA, continuous local intra-arterial nimodipine application; DCI, delayed cerebral ischemia; ERT, endovascular rescue therapy; GOSE, Glasgow Outcome Scale; iHTN, induced hypertension; INM, invasive neuromonitoring. (DOCX 14 kb) [file 12028_2020_1169_MOESM2_ESM.docx]

| **Variable** | **pre-INM (n=28)** | **post-INM (n=26)** | **p-value** |
| --- | --- | --- | --- |
| **GOSE result - no. (%)** |  |  |  |
| ***At 12 mo*** |  |  | 0.169 |
| Death | 3 (10.7) | 3 (11.5) |  |
| Vegetative state | 0 (0.0) | 0 (0.0) |  |
| Lower sever disability | 4 (14.3) | 0 (0.0) |  |
| Upper sever disability | 7 (25.0) | 7 (26.9) |  |
| Lower moderate disability | 2 (7.1) | 1 (3.8) |  |
| Upper moderate disability | 6 (21.0) | 1 (3.8) |  |
| Lower good recovery | 4 (14.3) | 7 (26.9) |  |
| Upper good recovery | 2 (7.1) | 7 (26.9) |  |
| Favorable outcome (5-8) - no. (%) | 14 (50.0) | 16 (61.6) | 0.253 |
| Shunt-dependency | 7 (25.0) | 5 (19.2) | 0.664 |
| **DCI diagnostics** |  |  |  |
| Silent infarction no. (%) | 8 (28.6) | 2 (7.7) | **0.048** |
| DCI-related infarction no. (%) | 12 (42.8) | 4 (23.1) | **0.027** |
| Overall mortality no. (%) | 3 (10.7) | 3 (11.5) | 0.928 |
| DCI-related mortality no. (%) | 3 (10.7) | 1 (3.8) | 0.336 |
| **DCI surveillance** |  |  |  |
| Imaging - mean ± SD |  |  |  |
| CTs | 9.8 ± 5.2 | 6.1 ± 4.0 | **0.003** |
| Angiography (incl. ERTs) | 2.3 ± 1.5 | 2.3 ± 1.8 | 0.349 |
| Patient transports | 12.2 ± 5.8 | 8.2 ± 5.3 | **0.011** |
| **DCI treatment** |  |  |  |
| iHTN - no. (%) | 27 (96.4) | 25 (96.2) | 0.977 |
| ERT - no. (%) | 10 (35.7) | 10 (38.5) | 0.704 |
| Angioplasty | 4 (14.3) | 3 (11.5) | 0.776 |
| Spasmolysis | 10 (35.7) | 7 (26.9) | 0.764 |
| CLINA | 1 (3.6) | 5 (19.2) | 0.067 |
| Pat. with multiple ERT | 5 (17.8) | 3 (11.5) | 0.514 |
| No. of ERT procedures | 18 ± 2.0 | 13 ± 2.0 | 0.682 |
